# Clinical- and Device-Related Factors Associated With Atrial High Rate Episodes in Patients With Dual-Chamber Pacemakers

**DOI:** 10.7759/cureus.86376

**Published:** 2025-06-19

**Authors:** Frank Jorge Valdez Baez, Elaine Nuñez Ayala, Juanico Cedano Ramirez, Cemirame Payan Jimenez, Catherine Merejo Peña, Laura Valdez de Leon, Warenny Montero Morillo, Evelina Severino Marte, Pedro Vidal Corniel Martinez

**Affiliations:** 1 Electrophysiology, Dominican Institute of Cardiology Association, Santo Domingo, DOM; 2 Surgery, Dominican Institute of Cardiology Association, Santo Domingo, DOM

**Keywords:** anticoagulation, arrhythmias, atrial fibrillation, atrial high-rate episodes, dual-chamber pacemakers, low-income countries, pacing strategy, subclinical atrial arrhythmias

## Abstract

Introduction

Atrial high-rate episodes (AHREs) detected by dual-chamber pacemakers may represent subclinical precursors of atrial fibrillation and thromboembolic events. However, the clinical-, electrocardiographic-, and device-related characteristics distinguishing patients with and without AHREs are not fully defined. This study aimed to compare these variables between both groups to identify those independently associated with the presence of AHREs.

Methods

This retrospective, single-center observational study included ambulatory patients with dual-chamber pacemakers evaluated between June and December 2024. Clinical, electrocardiographic, and device-related variables were compared between patients with and without AHREs. Bivariate analyses were conducted to identify significant differences, and multivariable logistic regression was used to determine variables independently associated with the presence of AHREs.

Results

A total of 450 individuals were included, of whom 185 (41.1%) exhibited AHREs, compared to 265 (58.9%) without AHREs, those affected were more frequently female, 116 (62.7%) versus 140 (52.8%), p = 0.037, and had a higher prevalence of prior atrial fibrillation or atrial tachycardia, 98 (53.0%) versus 68 (25.7%), p < 0.001. Ventricular pacing was slightly lower in the AHRE group (median 98% vs. 99%; p = 0.016), and atrial pacing mode with switch to dual-chamber (AAI-DDD) pacing mode was more common, 44 (23.8%) versus 32 (12.1%), p = 0.001. In multivariable analysis, prior atrial arrhythmias (OR 2.95; p < 0.001), follow-up ≥ 90 days (OR 4.14; p = 0.012), female sex (OR 1.63; p = 0.020), and AAI-DDD pacing (OR 1.92; p = 0.027) were independently associated with AHREs. The model demonstrated acceptable discrimination (area under the curve (AUC) = 0.70).

Conclusion

In this cohort, AHREs were observed in 41.1% of participants. Their occurrence was significantly associated with female sex, a prior history of atrial fibrillation or atrial tachycardia, a follow-up duration of 90 days or more, reduced ventricular pacing, and the use of the AAI-DDD pacing mode.

## Introduction

Atrial high-rate episodes (AHREs) are arrhythmias detected exclusively by cardiac implantable electronic devices (CIEDs), encompassing subclinical atrial fibrillation (AF), atrial flutter, and atrial tachycardia (AT). According to the 2023 guidelines from the ACC/AHA/ACCP/HRS (American College of Cardiology, American Heart Association, American College of Chest Physicians, and Heart Rhythm Society), AHREs are defined as atrial events that exceed a programmed detection threshold and require visual confirmation to differentiate true AF from artifacts or oversensing. Although no universal consensus has been established, the most accepted threshold is an atrial rate of ≥175 beats per minute lasting for ≥5-6 minutes - a temporal criterion that has been associated with an increased risk of future ischemic events [1].

The incidence of AHREs in patients with CIEDs varies widely depending on the detection criteria and the duration of follow-up. In the ASSERT (Asymptomatic AF and Stroke Evaluation in Pacemaker Patients and the AF Reduction Atrial Pacing Trial) study, the incidence was 10.1% at 30 months, increasing to 24.6% with extended follow-up [2]. Other studies have reported higher rates, with incidences reaching up to 55.9%, depending on monitoring duration and detection thresholds [3,4]. Among patients with dual-chamber pacemakers, an incidence exceeding 20% has been observed when applying a diagnostic threshold of ≥6 minutes at 175 bpm [5].

AHREs have been associated with various clinical-, electrocardiographic-, and device-related factors. Arterial hypertension and type 2 diabetes mellitus have been identified as independent risk factors [6]. From an electrocardiographic perspective, interatrial block (IAB), as well as increased P-wave duration (PWD) and dispersion, have been linked to a higher risk of AHREs [7,8]. Regarding pacemaker-related parameters, higher atrial and ventricular pacing burdens (≥50%) have been associated with an increased incidence of both AHREs and clinical AF [9]. Additionally, apical positioning of the ventricular lead and reduced P-wave amplitude as detected by the device (<2.45 mV) have been reported as predictors of AHRE onset and progression [10].

The clinical significance of AHREs remains a matter of ongoing debate. In 109 pacemaker patients (mean age 74 years, CHA₂DS₂-VASc 3.9), AHREs > 5 minutes occurred in 26% and silent ischemic brain lesions (IBLs) in 26%. IBLs correlated with age, higher risk scores, prior AF/stroke, and AHRE presence. AHREs independently predicted silent IBLs (HR 3.05 overall; HR 9.76 in patients without prior AF or stroke/TIA) [11]. A meta-analysis of four studies comprising 6788 patients found that anticoagulation was associated with a significant reduction in the risks of ischemic stroke (RR 0.66; 95% CI 0.49-0.90; p = 0.0084) and thromboembolic events (RR 0.70; 95% CI 0.55-0.89; p = 0.0037). Compared to AHREs not managed with anticoagulation, there was a significantly increased risk of major bleeding with anticoagulation (RR 1.57; 95% CI 1.23-2.00; p = 0.0002), while the risk of all-cause mortality was similar (RR 1.07; 95% CI 0.95-1.21; p = 0.2437) [12].

More recent data have yielded divergent results. The 2023 NOAH-AFNET 6 trial (Non-vitamin K Antagonist Oral Anticoagulants in Patients With Atrial High-Rate Episodes) showed no benefit of edoxaban in patients with AHREs ≥6 minutes and elevated CHA₂DS₂-VASc scores [13]. In contrast, the 2024 ARTESIA trial (Apixaban for the Reduction of Thromboembolism in Patients With Device-Detected Subclinical Atrial Fibrillation) demonstrated a significant reduction in stroke risk with apixaban (HR 0.63; p = 0.007), although this was accompanied by an increased risk of major bleeding (HR 1.80; p = 0.001) [14].

Current clinical guidelines recommend anticoagulation for AHREs lasting ≥24 hours in patients with a CHA₂DS₂-VASc score ≥2 (Class IIa) and consider it optional for episodes lasting between five minutes and 24 hours in patients with a CHA₂DS₂-VASc score ≥3 (Class IIb) [1].

Considering the variability in the reported prevalence of AHREs and the ambiguity regarding their clinical significance, particularly in low-income countries where data remain insufficient, this study seeks to identify clinical, electrocardiographic, and device-related factors associated with AHREs in patients with dual-chamber pacemakers in the Dominican Republic. The anticipated findings aim to enhance thromboembolic risk stratification, guide clinical decision-making, and support the development of more individualized therapeutic strategies for this population.

## Materials and methods

Study design

A retrospective observational study was conducted in the Department of Electrophysiology at the Asociación Dominican Institute of Cardiology (AIDC) in Santo Domingo, Dominican Republic. An analytical approach was employed to evaluate the association between clinical and electrocardiographic variables and the presence of AHREs. The study was conducted according to a predefined protocol, developed prior to data extraction.

A retrospective design was selected based on the availability of electronic medical records and previously collected data from patients with dual-chamber pacemakers. This approach enabled the assessment of AHRE frequency and associated factors without altering clinical practice or exposing patients to additional interventions. Moreover, it was well-suited to identify patterns within a real-world patient cohort while preserving standard medical care and minimizing external interference.

The AIDC is a specialized cardiology center with extensive experience in managing cardiac arrhythmias and implantable electronic devices. The institution maintains structured clinical records and is staffed by specialized personnel and equipped with advanced technology for the evaluation and follow-up of patients with dual-chamber pacemakers, offering an optimal setting for the conduct of this study.

Objective

This study aimed to determine the prevalence of AHREs in patients with dual-chamber pacemakers and to compare their clinical, electrocardiographic, and device programming characteristics in order to identify factors associated with their occurrence.

Study population

Patients who attended the specialized outpatient clinic of the Department of Electrophysiology between June and December 2024 were included in the study.

Sample and inclusion criteria

Individuals over 18 years of age, regardless of sex, who had undergone transvenous dual-chamber pacemaker implantation and had completed a minimum of 15 days of follow-up since the initial procedure were included in the study. Participants were consecutively recruited during routine outpatient follow-up visits between June and December 2024.

The required sample size was determined based on an anticipated prevalence of AHREs of 30%, a significance level of 0.05, a statistical power of 80%, and a moderate effect size (0.30). Considering that the study aimed to compare two groups using statistical tests, such as the independent samples Student’s t-test and the chi-square test, and assuming an asymmetric group distribution (2:1), the estimated total sample size was approximately 394 patients - 263 in the larger group and 131 in the smaller group. The calculation was performed using G*Power software (Franz Faul, Edgar Erdfelder, Albert-Georg Lang, and Axel Buchner, Heinrich Heine University Düsseldorf, Düsseldorf, Germany) to ensure sufficient statistical power to detect clinically meaningful differences between groups, accounting for the low prevalence of the condition of interest and the anticipated imbalance in group sizes.

Exclusion criteria

Patients were excluded if they exhibited a 100% AF (AF permanent) burden as detected by the pacemaker, or if they had an implantable cardioverter-defibrillator (ICD) or were undergoing cardiac resynchronization therapy (CRT), either with a pacemaker (CRT-P) or a defibrillator (CRT-D). Additional exclusion criteria included the presence of single-chamber pacemakers, devices programmed in VVI mode, or device-related complications such as infection, lead perforation, lead fracture, or lead dislodgement.

Exclusion criteria also included a history of congenital heart disease, hypertrophic cardiomyopathy, severe mitral or aortic valvular disease, valve replacement, treatment with antiarrhythmic agents or bronchodilators, and prior radiofrequency ablation for AF or AT.

Patients with recent major cardiovascular events (≤6 months), such as acute myocardial infarction (AMI), coronary artery bypass grafting (CABG), coronary stent implantation, or hospitalization for heart failure, were also excluded. Additionally, patients with systemic diseases or conditions that could compromise the validity of the study, such as chronic obstructive pulmonary disease (COPD), hyperthyroidism, or hypothyroidism requiring levothyroxine, were not included. Finally, patients whose pacemakers were implanted due to syncope unrelated to the study indication, or those with atrial lead artifacts that precluded accurate identification of atrial arrhythmias, were also excluded.

Data collection

During the clinical visit, the patient’s medical history was reviewed, followed by a physical examination and a six-lead surface electrocardiogram (ECG) using leads I, II, III, aVR, aVL, and aVF, instead of the conventional 12-lead ECG. The six-lead ECG was chosen because all electrocardiographic variables analyzed - paced QRS axis, IAB, and left bundle-branch block - are determined exclusively from frontal-plane information. The QRS axis is defined by limb-lead vectors; IAB is diagnosed by PWD ≥120 ms and biphasic morphology in the inferior leads (II, III, aVF); and LBBB is identified by a wide QRS ≥120 ms with limb-lead morphology compatible with leftward depolarization, findings that do not require precordial leads. Therefore, using six leads ensured reliable measurement of the variables relevant to our study while facilitating rapid acquisition during routine pacemaker follow-up.

Demographic variables, including age (in years) and sex, were recorded, along with clinical history variables such as hypertension, diabetes mellitus, stroke, AMI, and prior non-permanent AF or atrial tachycardia (AT), documented through previous surface ECGs or ECG monitoring. Additional variables included heart failure, left bundle branch block, IAB, and smoking status. All variables were assessed as binary outcomes (present or absent).

These variables were also used to calculate the CHA₂DS₂-VASc score, an established tool for estimating thromboembolic risk. The score assigns one point for the presence of congestive heart failure, hypertension, diabetes mellitus, vascular disease (including prior myocardial infarction, peripheral arterial disease, or aortic plaque), age between 65 and 74 years, and female sex. Two points are assigned for age ≥75 years and a history of stroke, transient ischemic attack (TIA), or systemic thromboembolism. The total score ranges from zero to nine, with values ≥3 indicating a high thromboembolic risk.

Pharmacological treatment was documented for each patient, including the use of angiotensin-converting enzyme inhibitors (ACEIs), angiotensin II receptor blockers (ARBs), angiotensin receptor-neprilysin inhibitors (ARNIs), beta-blockers, diuretics, sodium-glucose cotransporter 2 inhibitors (SGLT2i), anticoagulants, and antiplatelet agents. In addition, systolic blood pressure (SBP) and diastolic blood pressure (DBP) were recorded in mmHg.

Device interrogation was performed using the manufacturer-specific pacemaker programmer. AHREs were identified and validated by experienced electrophysiologists based on intracardiac electrogram (EGM) analysis. All relevant clinical-, electrocardiographic-, and device-related data were systematically extracted using a structured case report form designed for this study.

Specific pacemaker-related data were collected, including the indication and type of implantation, time since implantation or follow-up (in days), device manufacturer, pacing mode, atrial EGM amplitude (in millivolts), and the percentages of atrial and ventricular pacing. The presence of AHREs was documented. Additionally, electrocardiographic parameters, such as the axis of the paced QRS complex (in degrees), were assessed using a six-lead surface ECG.

Patients with missing or incomplete clinical, device-related, or electrocardiographic records were excluded from the analysis to maintain data integrity.

AHRE validation

AHRE detection was confirmed through expert visual validation of intracardiac EGMs by experienced electrophysiologists. Only episodes consistent with atrial tachyarrhythmia morphology, defined by rapid and organized atrial activity with a regular ventricular response, in the absence of noise or signal distortion, were considered valid. Episodes suggestive of electrical interference, oversensing, far-field sensing, or myopotential artifacts were systematically excluded. In cases of diagnostic uncertainty or disagreement regarding episode classification, a second electrophysiologist from the department was consulted. Episodes for which disagreement persisted after review by both specialists were excluded from the analysis to ensure high-quality event adjudication and consistency across the dataset.

AHREs were defined as atrial events exceeding a programmed detection threshold pre-established by each device manufacturer and requiring visual confirmation by electrophysiologists to differentiate true AF from artifacts or oversensing. These thresholds varied by device: >175 bpm for Medtronic (Minneapolis, MN), >180 bpm for Boston Scientific, Marlborough, MA,>220 bpm for St. Jude Medical (SJM)/Abbott (Saint Paul, MN), and 180-220 bpm for Biotronik (Berlin, Germany), depending on the model. The outcome was treated as a binary variable (present/absent), and neither the duration nor the temporal burden of AHREs was recorded or analyzed, as prognostic evaluation was not the objective of this study.

Operationalization of variables

For statistical analysis, quantitative variables were categorized as follows: age was grouped into <70 and ≥70 years, as well as <65 and ≥65 years; the percentages of ventricular and atrial pacing were dichotomized as <50% and ≥50%; atrial EGM amplitude was classified as <1 mV and ≥1 mV; and the QRS axis was categorized as apical (-30° to -90°) or basal (-30° to +120°).

Additionally, the number of medications prescribed was categorized as <3 or ≥3 drugs; the CHA₂DS₂-VASc score was dichotomized as <3 or ≥3 points; and follow-up duration was classified both in annual intervals and dichotomously as <90 or ≥90 days. Finally, device-related variables were categorized according to type of implantation (initial implant vs. generator replacement), pacing mode (DDD pacing mode (DDD) vs. atrial pacing mode with switch to dual-chamber(AAI-DDD)), and pacemaker indication (atrioventricular block vs. sick sinus syndrome (SSS)).

Continuous variables were dichotomized using clinically validated cut-offs drawn from previous AHRE studies and guideline thresholds to facilitate comparison with the existing literature and enhance bedside interpretability. Specifically, ≥50% pacing was chosen because several reports link ventricular or atrial pacing burdens above this level to higher AHRE incidence; a CHA₂DS₂-VASc score ≥3 denotes high thromboembolic risk; and ≥65/≥75 years reflect age bands commonly applied in arrhythmia risk stratification. Each variable was first examined in its continuous form, yielding results consistent with the dichotomized analysis, and only then recoded for multivariable modelling, so any loss of statistical power or granularity was minimal and did not alter the direction or significance of associations.

Paced QRS axis was grouped as apical (-30° to -90°) versus basal (-30° to +120°) because these frontal-plane ranges provide a practical surrogate for right-ventricular lead position when operative fluoroscopy reports are inconsistent or unavailable. An inferior-leftward axis (apical range) typically reflects apical pacing, whereas a more superior or rightward axis (basal range) suggests septal or outflow-tract pacing; thus, this dichotomy captures clinically meaningful differences in ventricular activation without fragmenting the cohort into very small subgroups. Axes falling outside these limits were rare and excluded to maintain analytical consistency. 

Statistical analysis

For the purposes of data analysis, the sample was divided into two groups: patients with AHREs and those without.

Continuous variables were expressed as mean ± standard deviation or as median with interquartile range (IQR), according to the distribution of the data assessed by the Kolmogorov-Smirnov test. Between-group comparisons were performed using the Student’s t-test for normally distributed variables and the Mann-Whitney U test for non-normally distributed variables. Categorical variables were presented as absolute frequencies and percentages and were compared using Pearson’s chi-square test or Fisher’s exact test, as appropriate, with the latter applied when the expected frequency in any cell was less than five.

A bivariate logistic regression analysis was conducted to identify factors associated with the presence of AHREs, and results were expressed as odds ratios (ORs) with corresponding 95% confidence intervals (95% CI). Variables included in the multivariable model were selected based on statistical significance in the univariate analyses (p < 0.05) and clinical relevance. Prior to inclusion, all candidate variables were assessed for multicollinearity using variance inflation factors (VIFs). A conservative VIF threshold of >2.5 was applied, in line with recommendations for multivariable models involving moderate sample sizes and conceptually related predictors. The multivariable logistic regression model’s discrimination was evaluated by the ROC curve, with the AUC quantifying performance (AUC closer to one indicates better discrimination). Bootstrap resampling (1000 iterations) assessed model stability. Model fit was measured by -2 log likelihood, and explanatory power by Cox and Snell, Nagelkerke, and McFadden pseudo-R² statistics. An optimal probability cut-off balanced sensitivity and specificity, supporting clinical risk stratification. Calibration was assessed with the Hosmer-Lemeshow goodness-of-fit test, using deciles of predicted probability to compare observed versus expected event rates.

Collinearity was assessed using VIF calculated from the initial full logistic model; AAI-DDD mode and sick-sinus syndrome exhibited VIFs of 1.47 and 1.92, respectively, while low ventricular pacing (<50%) showed a VIF of 2.08. Because the latter overlapped conceptually with these predictors and did not improve model fit (AIC and -2 log likelihood unchanged), it was excluded to preserve parsimony, leaving all final covariates with VIFs < 2.

It is worth noting that, based on the Kolmogorov-Smirnov test used to assess the normality of continuous variables, only SBP exhibited a normal distribution in both groups. Consequently, it was the only variable analyzed using the independent samples Student’s t-test. All other continuous variables were compared using the Mann-Whitney U test.

QRS axis data were available for all patients in the study. However, only two axis categories - normal axis and left axis deviation - were included in the analysis. Patients whose QRS axis did not fall within these predefined categories (e.g., right axis deviation or extreme axis) were excluded from the axis-based subgroup comparisons to maintain analytical consistency.

Data were processed using IBM SPSS Statistics for Windows, version 23.0 (IBM Corp., Armonk, NY). Sample size requirements, statistical power, and effect size estimations were performed as described in the corresponding subsection.

Ethical considerations

This study was approved by the Ethics Committee of the AIDC under approval number AIDC-CE-2025-001 and was conducted in accordance with the ethical principles of the Declaration of Helsinki. Analyses were performed without discrimination based on sex. Given the observational and retrospective nature of the study, informed consent was not required, as only previously recorded data were used and no direct interaction with patients occurred. All data were handled with strict confidentiality and anonymized in compliance with international data protection regulations.

The authors declare no conflicts of interest and report no external funding that could have influenced the outcomes of this study. All data and findings generated are fully presented within the manuscript. Artificial intelligence assistance was limited to improving the linguistic clarity of the text through the use of ChatGPT (GPT-4o, OpenAI, San Francisco, CA); the model did not contribute to the interpretation of the results or the development of the scientific content, which remained entirely under the authors’ responsibility.

## Results

The initial cohort consisted of 997 patients. Of these, 484 were excluded for meeting at least one of the predefined exclusion criteria, and 63 were removed due to duplication in the database. Consequently, the final analytical sample comprised 450 patients with dual-chamber pacemakers, of whom 185 (41.1%) exhibited AHREs, while 265 (58.9%) did not.

Throughout this manuscript, the term independently associated is reserved exclusively for variables that retained statistical significance in the multivariable logistic-regression model.

In the subsequent analysis, Table 1 shows that the only continuous variable with a statistically significant difference between groups was the percentage of ventricular pacing, which was slightly lower in patients with AHREs (median 98% (IQR 12-100)) compared to those without AHREs (99% (IQR 63-100); p = 0.016). In contrast, no significant differences were found between groups regarding age (median 80 years), systolic and DBP, CHA₂DS₂-VASc score, number of medications, follow-up duration, atrial EGM amplitude, or atrial pacing percentage (all p > 0.05).

**Table 1:** Comparison of Continuous Clinical and Device-Related Variables Between Patients With and Without Atrial High-Rate Episodes (AHREs) Results of the comparison between patients with and without AHREs regarding continuous clinical and device-related variables are presented. Variables include demographic data, blood pressure, clinical risk scores, medication count, follow-up duration, atrial electrogram amplitude, and atrial and ventricular pacing percentages. Continuous variables with non-normal distribution are presented as median (25th-75th percentiles) and were compared using the Mann-Whitney U test. Systolic blood pressure, which showed a normal distribution in both groups, is presented as mean ± standard deviation (SD) and was compared using the independent samples Student’s t-test. Statistical significance was defined as two-tailed p < 0.05. %: percentage; AHREs: atrial high-rate episodes; CHA₂DS₂-VASc: congestive heart failure, hypertension, age ≥75 (two points), diabetes mellitus, stroke/TIA/thromboembolism (two points), vascular disease, age 65-74, sex category (female); DBP: diastolic blood pressure; EGM: electrogram; mV: millivolts; SBP: systolic blood pressure; SD: standard deviation

Variables Continuous	No AHREs (n = 265)		With AHREs (n = 185)		
Demographic/blood pressure	Median	p25-p75	Median	p25-p75	p
Age (years)	80	75-89	80	76-89	0.611
SBP (mmHg) (mean ± SD)	142.42	±24.64	141.71	±22.08	0.754
DBP (mmHg)	75	69-82	77	69-84	0.151
Clinical scores					
CHA₂DS₂-VASc score	3	2-4	3	3-4	0.799
Number of medications	3	1-4	3	2-3	0.441
Device-related variables					
Follow-up (days)	2008	(673-3216)	2096	(896-3230)	0.454
Atrial EGM amplitude (mV)	2.8	(2-4.7)	2.8	(1.7-4.1)	0.305
Atrial pacing (%)	6.4%	(1-29)	5%	(1-23)	0.399
Ventricular pacing (%)	99%	(63-100)	98%	(12-100)	0.016

Similarly, categorical variables presented in Table 2 reveal a significantly higher proportion of female patients in the AHRE group compared to those without AHREs (62.7% vs. 52.8%; p = 0.037). However, no significant differences were detected in age group distribution, systolic or DBP categories, or electrocardiographic parameters, including QRS axis orientation, presence of IAB, and left bundle branch block (all p > 0.05).

**Table 2:** Comparison of Clinical and Electrocardiographic Variables According to the Presence of Atrial High-Rate Episodes (AHREs) Results of the comparison between patients with and without atrial high-rate episodes (AHREs), based on clinical and electrocardiographic variables. QRS axis data were available for 380 patients. Categorical variables are expressed as absolute frequencies and percentages and were analyzed using Pearson’s chi-square test. All statistical comparisons were two-sided, and a p-value < 0.05 was considered statistically significant. %: percentage; AHREs: atrial high-rate episodes; DBP: diastolic blood pressure; mmHg: millimeters of mercury; n: number; QRS: QRS complex; SBP: systolic blood pressure

Variables	No AHREs (n = 265)		With AHREs (n = 185)		
Demographic/blood pressure	n	%	n	%	p
Sex					
Male	125	47.17%	69	37.30%	0.037
Female	140	52.83%	116	62.70%	
Age group (years)					
≥75	147	55.47%	106	57.30%	0.701
<75	118	44.53%	79	42.70%	
≥65	218	82.26%	158	85.41%	0.376
<65	47	17.74%	27	14.59%	
SBP (mmHg)					
<140	129	48.68%	83	44.86%	0.425
≥140	136	51.32%	102	55.14%	
DBP (mmHg)					
<90	212	80.00%	147	79.46%	0.888
≥90	53	20.00%	38	20.54%	
Electrocardiographic					
QRS axis (n = 380)					
Basal	62	27.31%	41	26.80%	0.912
Apical	165	72.69%	112	73.20%	
Total	227	100%	153	100.00%	
Interatrial block					
No	256	96.60%	176	95.14%	0.434
Yes	9	03.40%	9	04.86%	
Left bundle branch block					
Yes	11	04.15%	6	03.24%	0.619
No	254	95.85%	179	96.76%	

Building upon these clinical characteristics, Table 3 highlights a significantly higher prevalence of prior AF or AT among patients with AHREs compared to those without (53.0% vs. 25.7%; p < 0.001). In contrast, no significant differences were observed in other comorbidities such as hypertension, diabetes mellitus, heart failure with reduced ejection fraction, vascular disease, or history of stroke (all p > 0.05). Polypharmacy (≥3 drugs) did not differ significantly between groups (50.19% vs. 53.5%; p = 0.487).

**Table 3:** Comparison of Comorbidities and Therapeutic Variables According to the Presence of Atrial High-Rate Episodes (AHREs) Results of the comparison between patients with and without atrial high-rate episodes (AHREs), based on comorbidities, pharmacological treatment, and clinical scores. Categorical variables are presented as absolute frequencies and percentages and were compared using Pearson’s chi-square test. All statistical comparisons were two-sided, and a p-value < 0.05 was considered statistically significant. %: percentage; ACEIs: angiotensin-converting enzyme inhibitors; AF: atrial fibrillation; AHREs: atrial high-rate episodes; ARBs: angiotensin receptor blockers; ARNIs: angiotensin receptor-neprilysin inhibitors; AT: atrial tachycardia; CHA₂DS₂-VASc: thromboembolic risk score; CHF: congestive heart failure; LVEF: left ventricular ejection fraction; n: number; SD: standard deviation; SGLT2: sodium-glucose cotransporter-2

Variables	No AHREs (n = 265)		With AHREs (n = 185)		
Comorbidities	n	%	n	%	p
Arterial hypertension	234	88.30%	167	90.27%	0.510
Diabetes mellitus	79	29.81%	45	24.32%	0.200
Prior AF-AT	68	25.66%	98	52.97%	<0.001
CHF-LVEF ≤ 40%	39	14.72%	21	11.35%	0.301
Vascular disease	29	10.94%	20	10.81%	0.965
Stroke	11	04.15%	15	08.11%	0.077
Smoker	4	01.51%	2	01.08%	0.697
CHA₂DS₂-VASc ≥ 3	195	73.58%	139	75.14%	0.711
Therapeutic					
ACEIs/ARBs/ARNIs	184	69.43%	123	66.49%	0.509
Calcium channel blockers	125	47.17%	99	53.51%	0.185
Loop diuretics	112	42.26%	90	48.65%	0.180
Antiplatelet agents	73	27.55%	51	27.57%	0.996
Beta-blockers	65	24.53%	52	28.11%	0.394
Statins	49	18.49%	26	14.05%	0.214
Aldosterone antagonists	23	08.68%	15	08.11%	0.830
Anticoagulants	20	07.55%	22	11.89%	0.119
SGLT2 inhibitors	16	06.04%	7	03.78%	0.285
Polypharmacy ≥ 3	133	50.19%	99	53.51%	0.487

Complementing these findings, Table 4 presents several device-related categorical variables that were significantly associated with the presence of AHREs. Specifically, patients with AHREs were more frequently implanted for SSS (27.0% vs. 16.6%; p = 0.007) and less frequently for atrioventricular block (73.0% vs. 83.4%). Furthermore, the AAI-DDD pacing mode was more common in the AHRE group (23.8% vs. 12.1%; p = 0.001), as was ventricular pacing below 50% (32.4% vs. 21.9%; p = 0.012). The distribution of pacemaker manufacturers also differed significantly overall (p = 0.001), with fewer Biotronik devices (3.2% vs. 9.4%; p = 0.018) and more SJM devices (8.1% vs. 1.9%; p = 0.003) among patients with AHREs. Additionally, a follow-up duration of ≥90 days was more frequent in this group (97.8% vs. 88.7%; p < 0.001). No significant differences were found in type of implant (p = 0.722), atrial EGM amplitude <1 mV (14.6% vs. 10.9%; p = 0.248), atrial pacing ≥50% (10.8% vs. 15.5%; p = 0.155), or follow-up duration categorized by years (p = 0.055).

**Table 4:** Comparison of Pacemaker-Related Variables According to the Presence of Atrial High-Rate Episodes (AHREs) Results of the comparison between patients with and without atrial high-rate episodes (AHREs), based on device characteristics, electrical parameters, and follow-up duration. Categorical variables are expressed as absolute frequencies and percentages and were analyzed using Pearson’s chi-square test. All statistical comparisons were two-sided, and a p-value < 0.05 was considered statistically significant. %: percentage; AAI-DDD: atrial pacing mode with switch to dual-chamber; AHRE: atrial high-rate episodes; AV: atrioventricular; DDD: DDD pacing mode; EGM: electrogram; mV: millivolts; n: number; SJM: St. Jude Medical

Variables	No AHREs (n = 265)		With AHREs (n = 185)		
Categorical variables	n	%	n	%	p
Type of implant					
First implant	201	75.85%	143	77.30%	0.722
Replacement	64	24.15%	42	22.70%	
Indication					
AV block	221	83.40%	135	72.97%	0.007
Sick sinus syndrome	44	16.60%	50	27.03%	
Manufacturer					0.001
Boston Scientific Corp.	150	56.60%	113	61.08%	0.394
Medtronic	85	32.08%	51	27.57%	0.357
Biotronik	25	9.43%	6	3.24%	0.018
SJM	5	1.89%	15	8.11%	0.003
Pacing mode					
DDD	233	87.92%	141	76.22%	0.001
AAI-DDD	32	12.08%	44	23.78%	
Atrial EGM amplitude (mV)					
<1	29	10.94%	27	14.59%	0.248
≥1	236	89.06%	158	85.41%	
Atrial pacing (%)					
<50	224	84.53%	165	89.19%	0.155
≥50	41	15.47%	20	10.81%	
Ventricular pacing (%)					
<50	58	21.89%	60	32.43%	0.012
≥50	207	78.11%	125	67.57%	
Follow-up duration (days)					
<90	30	11.32%	4	2.16%	<0.001
≥90	235	88.68%	181	97.84%	
Follow-up duration (years)					0.055
1	51	19.25%	20	10.81%	-
2	16	6.04%	17	9.19%	
3	18	6.79%	21	11.35%	
4	18	6.79%	15	8.11%	
≥5	162	61.13%	112	60.54%	

Building on these findings, Table 5 presents the results of bivariate and multivariable logistic regression analyses assessing factors associated with AHREs. In the bivariate analysis, follow-up duration ≥90 days (OR 5.78, p = 0.001), SJM device manufacturer (OR 4.58, p = 0.003), prior AF or AT (OR 3.26, p < 0.001), AAI-DDD pacing mode (OR 2.27, p = 0.001), SSS (OR 1.86, p = 0.008), ventricular pacing <50% (OR 1.71, p = 0.013), and female sex (OR 1.50, p = 0.038) were all significantly associated with AHREs. Conversely, the Biotronik manufacturer was inversely associated with AHREs (OR 0.32, p = 0.014), while prior cerebrovascular accident or TIA showed a non-significant trend toward association (OR 2.04, p = 0.082).

**Table 5:** Bivariate and Multivariable Logistic Regression Analysis for the Presence of Atrial High-Rate Episodes (AHREs) Results of the bivariate and multivariable logistic regression analysis to identify variables associated with the presence of atrial high-rate episodes (AHREs). P-values, odds ratios (OR), and their corresponding 95% confidence intervals (95% CI) are presented. The multivariable model included variables selected based on their clinical relevance and/or statistical significance in the bivariate analysis. All statistical comparisons were two-sided, and a p-value < 0.05 was considered statistically significant. %: percentage; AAI-DDD: atrial pacing with switch to dual-chamber mode; AF: atrial fibrillation; AHREs: atrial high-rate episodes; AT: atrial tachycardia; CI: confidence interval; OR: odds ratio; p: statistical significance level; SJM: St. Jude Medical

Logistic Regression Analysis	p	Odds Ratio	95% CI
Bivariate logistic regression			
Follow-up duration ≥ 90 days	0.001	5.78	2.00-16.69
SJM manufacturer	0.003	4.58	1.63 -12.85
Prior AF-AT	<0.001	3.26	2.19-4.86
AAI-DDD mode	0.001	2.27	1.38-3.75
Stroke	0.082	2.04	0.91-4.54
Sick sinus syndrome	0.008	1.86	1.18-2.94
Ventricular pacing < 50%	0.013	1.71	1.12-2.62
Female sex	0.038	1.50	1.02-2.2
Biotronik manufacturer	0.014	0.32	0.12-0.80
Multivariable logistic regression			
Follow-up ≥ 90 days	0.012	4.14	1.37-12.53
Prior AF-AT	<0.001	2.95	1.95-4.46
AAI-DDD mode	0.027	1.92	1.08-3.42
Female sex	0.02	1.63	1.08-2.45
Sick sinus syndrome	0.159	1.47	0.86-2.52

In the multivariable model, follow-up duration ≥90 days (OR 4.14, p = 0.012), prior AF-AT (OR 2.95, p < 0.001), AAI-DDD pacing mode (OR 1.92, p = 0.027), and female sex (OR 1.63, p = 0.020) remained independent predictors. SSS did not reach statistical significance (OR 1.47, p = 0.159).

**Figure 1:**
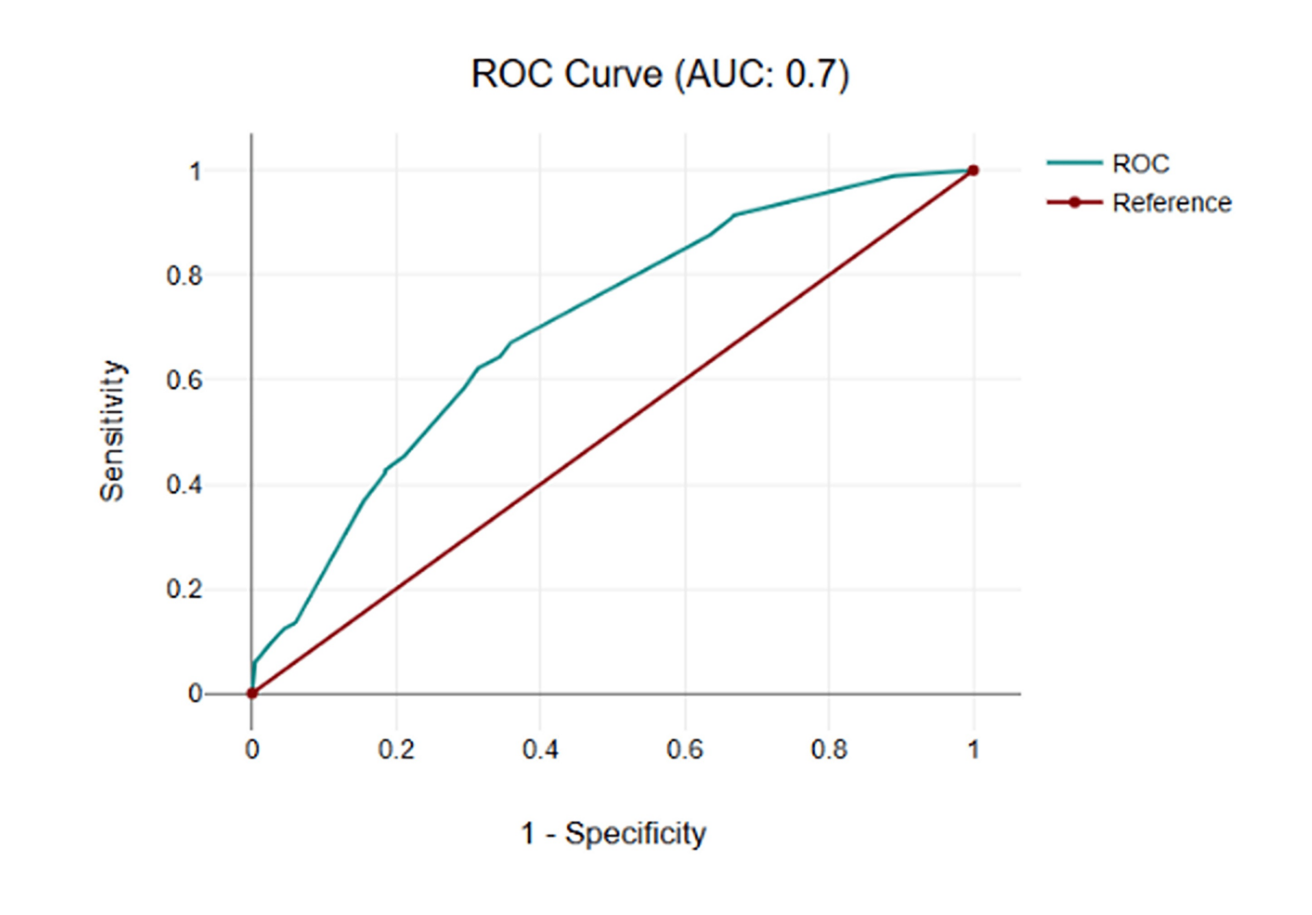
ROC Curve for the Multivariable Logistic Regression Model Predicting Atrial High-Rate Episodes (AHREs) The ROC curve displays the discriminatory performance of the multivariable logistic regression model predicting AHREs, with an AUC of 0.70 (95% CI, 0.66-0.75). Details of the model variables and statistics are provided in Table 5. AUC: area under the curve; AHREs: atrial high-rate episodes; ROC: receiver operating characteristic

The multivariable logistic regression model demonstrated acceptable discrimination for predicting AHREs, with an AUC of 0.70 (95% CI 0.66-0.75), estimated via bootstrap resampling (1000 iterations), confirming model robustness (Figure 1). The optimal probability cut-off of 0.24 balanced sensitivity (74%) and specificity (89%) (Youden’s index), providing clinical utility for risk stratification in patients with dual-chamber pacemakers.

Model fit was adequate, with a -2 log likelihood of 550.07. The pseudo-R² values (Cox and Snell = 0.12, Nagelkerke = 0.17, McFadden = 0.10) indicate low-to-moderate explanatory power, which is customary for logistic regression applied to clinical data. While the model offers useful discrimination (AUC = 0.70), its explained variance is modest and should be interpreted accordingly. Calibration was also satisfactory: the Hosmer-Lemeshow goodness-of-fit test with 10 risk deciles produced χ² = 7.9 (df = 8, p = 0.45), demonstrating near-perfect alignment between predicted and observed probabilities over the entire risk spectrum.

In summary, this study identified key clinical and device-related factors independently associated with the presence of AHREs in patients with dual-chamber pacemakers. The multivariable model demonstrated moderate predictive accuracy and robustness, supporting its potential utility in clinical risk stratification. These findings provide a foundation for further research to refine predictive tools and guide personalized management in this patient population.

## Discussion

In this study, 41.1% of patients with dual-chamber pacemakers exhibited AHREs, which were significantly associated with a prior history of atrial arrhythmias, AAI-DDD pacing mode, lower ventricular pacing burden, and a follow-up duration of ≥90 days. These associations suggest that AHREs may represent an early marker of atrial electrical or structural dysfunction and could serve as a potential precursor to clinical AF and related thromboembolic events.

Prevalence

Published series reports a heterogeneous prevalence of AHREs, varying from 13% to 65% and reaching 88.6% among patients with a prior diagnosis of AF [15-17]. A contemporary Indian cohort documented an incidence of 17% after dual-chamber pacemaker implantation, underscoring the influence of population characteristics and follow-up intensity on detection rates [18].

Subclinical AF is associated with more than a two-fold prevalence of previous clinical AF (HR 3.521; p < 0.001) [19], while prolonged AHREs occur significantly more often in patients with paroxysmal supraventricular tachycardia (43% vs. 14%; p < 0.001) [4]. Collectively, these findings support the interpretation of AHREs as early manifestations of atrial dysfunction and potential precursors to overt AF.

An adjusted OR of 2.95 indicates that patients with a prior history of AT, flutter, or fibrillation are nearly three times more likely to develop AHREs, underscoring the need for closer device interrogations, early remote monitoring, and timely reassessment of anticoagulation in this subgroup. Because these arrhythmias often coexist, interconvert, and confer similar embolic risk when prolonged, we combined prior AT and AF into a single composite predictor to minimize redundancy and collinearity while capturing the shared pathological continuum; incidental AHRE detection should therefore trigger an active search for overt AF and, when appropriate, early initiation of preventive anticoagulation.

The pathogenesis of AHREs likely reflects combined electrical and structural atrial remodeling, namely, refractory-period shortening, fibrosis, and dilatation [20]. Shared inflammatory pathways further promote progression from subclinical AHREs to symptomatic AF [21,22].

Inflammatory biomarkers were not available in our retrospective dataset; high-sensitivity C-reactive protein, interleukin-6, and the monocyte-to-HDL ratio were not routinely recorded, precluding analysis. Nevertheless, emerging evidence suggests that systemic inflammation and profibrotic signaling, reflected by elevated hs-CRP, IL-6, monocyte/high-density lipoprotein (HDL) ratio, and galectin-3, may link atrial remodeling to device-detected AHREs and their progression to clinical AF.

Our study did not aim to assess AHREs as prognostic indicators nor to examine their association with future clinical events. Therefore, we used a simplified binary classification (presence or absence of AHREs), irrespective of duration, and based solely on device detection and visual confirmation, without applying prognostic duration thresholds (≥5-6 minutes, ≥6 minutes, ≥30 seconds, ≥24 hours).

Our cohort displays characteristics that constrain external generalization. Participants were elderly (median 80 years; IQR 76-89) and highly comorbid. Most pacemakers were implanted for atrioventricular block rather than SSS. All patients were treated in a single public center in the Dominican Republic, where remote monitoring and direct oral anticoagulants are less accessible than in high-income settings. This combination of advanced age, substantial vascular risk, and resource-limited context should be considered when extrapolating our results to younger populations, single-chamber devices, or healthcare systems with universal remote monitoring and wider anticoagulation availability.

Ischemic risk scores

Taken together, AHREs may serve as early markers of atrial dysfunction and reflect a significant arrhythmic burden; however, their clinical predictive value appears to vary depending on the patient’s risk profile and the presence of underlying electrical or structural remodeling. Due to their association with thromboembolic events, several studies have explored the utility of conventional risk stratification tools, such as the CHA₂DS₂-VASc score, in patients with subclinical atrial arrhythmias. In cohorts undergoing CRT, elevated CHA₂DS₂-VASc scores have been associated with an increased risk of AHREs lasting between six and 24 hours [23]. Nonetheless, other investigations have questioned the predictive capacity of this score in this population [19], underscoring the need for more specific and sensitive risk markers in device-detected arrhythmias.

Recent evidence has emphasized the importance of underlying coronary artery disease (CAD) as a significant contributor to the development and prognosis of both AF and AHREs in patients with CIEDs. CAD may promote atrial ischemia, fibrosis, and autonomic imbalance, thereby increasing arrhythmogenic potential and facilitating subclinical atrial arrhythmias. Moreover, the coexistence of CAD has been associated with a higher risk of stroke and all-cause mortality in patients with AHREs, underscoring the need for systematic assessment, especially in individuals with high suspicion or established cardiovascular risk factors [24-28].

In our cohort, CHA₂DS₂-VASc did not discriminate between patients with and without AHREs; this finding should be interpreted cautiously and validated in larger, independent populations before any conclusion about its utility in device-detected arrhythmias can be drawn.

Pacemaker programming

In our cohort, AAI-DDD pacing was significantly associated with a higher incidence of AHREs (OR: 1.92; p = 0.027), as was pacemaker implantation for SSS (OR: 1.86; p = 0.008). These findings support the hypothesis that sinus node dysfunction represents a vulnerable substrate for the development of subclinical atrial arrhythmias [29], where electrocardiographic markers such as prolonged PWD have also been shown to independently predict AHRE occurrence.

AAI-DDD mode prioritizes atrial sensing/pacing and switches to ventricular support only when AV block is detected, making it the default programming for patients with SSS who retain intact AV conduction. Accordingly, a higher prevalence of AAI-DDD in the AHRE group, together with their lower ventricular pacing burden, likely reflects an underlying substrate of sinus-node dysfunction with preserved AV conduction rather than a direct causal effect of the mode itself; except in cases of spontaneous or therapy-induced AV block (e.g., anti-arrhythmic drugs, AV-node ablation), ventricular pacing is expected to remain low in this population.

No causal relationship is implied between AAI-DDD mode and AHREs; device programming is dictated by the clinical indication, and the observed association likely reflects patient selection, particularly among individuals with sinus-node dysfunction, rather than a direct effect of the pacing mode itself.

Recent studies have further linked inflammatory and oxidative stress markers, such as an elevated monocyte-to-HDL cholesterol ratio, to an increased risk of AHREs in patients with dual-chamber pacemakers and underlying sinus node dysfunction [30]. Additionally, anatomical abnormalities like IAB have been implicated in the pathogenesis of atrial arrhythmias in this population [31].

In terms of pacing modality, a comparative study demonstrated that closed-loop stimulation (CLS) significantly reduced the incidence of AHREs lasting ≥6 minutes compared to accelerometer-based DDDR pacing in patients with SSS [32]. These findings suggest that physiologically responsive pacing strategies may have a role in modulating atrial electrical stability.

Additionally, a lower ventricular pacing burden (<50%) was also significantly associated with an increased likelihood of AHREs (OR: 1.71; p = 0.013). However, evidence regarding the impact of ventricular pacing suppression remains mixed. A 2011 meta-analysis of 21 randomized trials involving 8336 patients found that minimizing ventricular pacing to <10% did not significantly reduce the progression to clinical AF [16].

Frequent right-ventricular pacing, particularly from apical or septal positions, widens the paced QRS complex, induces mechanical dyssynchrony, elevates filling pressures, and leads to bi-atrial stretch. This hemodynamic stress, compounded by retrograde ventriculo-atrial conduction and repetitive AV-node activation, fosters the electrical and structural remodeling that precipitates AHREs. Pacing modes or algorithms that raise average heart rate or yield ventricular-pacing burdens ≥50% intensify this substrate, whereas more physiological approaches (His-bundle or left-bundle-branch-area pacing) and AV-hysteresis or adaptive AV-delay programming can attenuate it. A markedly prolonged paced QRS duration, therefore, serves as an indirect marker of this arrhythmogenic milieu.

Taken together, these data suggest that atrial interstitial fibrosis in patients with sinus node dysfunction plays a central role in the initiation of AHREs and clinical AF. This arrhythmogenic process may be exacerbated by interatrial conduction delay and unnecessary right ventricular pacing, both of which contribute to mechanical dyssynchrony, hemodynamic impairment, and progressive atrial remodeling. Therefore, a lower percentage of unnecessary ventricular pacing was associated with a smaller arrhythmic burden in our cohort; prospective trials are needed to determine whether pacing reduction leads to fewer clinical arrhythmias.

Female sex

In our study, female sex was independently associated with the presence of AHREs, a finding that contrasts with most prior investigations, which have generally failed to identify a significant relationship between sex and AHRE occurrence. Notably, one isolated study reported an association with male sex, specifically about AHREs of intermediate duration (≥6 minutes and <24 hours) [33].

Multiple non-hormonal factors may explain the female-sex signal [34]: women more often require AAI-DDD pacing for sick-sinus syndrome, resulting in lower ventricular-pacing burdens linked to AHREs [35]; electro-anatomic maps show greater fibrosis and reduced voltage, especially in paroxysmal AF, favoring re-entry [36,37]; smaller atria experience higher wall stress during pacing-induced dyssynchrony [38]; autonomic patterns (higher vagal tone, greater sympathetic reactivity) further shorten refractoriness [39]; and post-menopausal rises in hs-CRP, IL-6 and other profibrotic mediators accelerate remodeling [40,41]. Women also exhibit higher AF recurrence after ablation, supporting an intrinsically arrhythmogenic substrate [42]. These converging data underpin our observation, though larger sex-stratified cohorts are needed for confirmation.

These divergent findings underscore the need for further research to clarify whether sex-related differences influence the incidence, arrhythmic burden, or clinical implications of AHREs. Future studies should also explore potential interactions with hormonal status, atrial structural remodeling, and device programming parameters that may contribute to sex-specific arrhythmogenic risk.

Follow-up

In our cohort, a follow-up duration of ≥90 days was independently associated with the detection of AHREs (OR: 4.14; p = 0.012), reinforcing the notion that extended monitoring increases the likelihood of capturing these episodes. This observation aligns with previous studies, including one reporting the onset of AHREs (>5 minutes) in 60% of patients after three months and in 65% after 18 months of follow-up [18]. Prospective investigations have typically adopted observation periods of at least six months to assess AHRE incidence [4,43]. For instance, one study found that 30.3% of patients developed AHREs during a six-month follow-up period [4].

Collectively, the available evidence suggests that AHREs may arise as early as the first month following CIED implantation and continue to emerge over subsequent years. However, definitions of “new onset” frequently exclude episodes detected within the first one to three months, highlighting the lack of standardized temporal criteria across studies.

Although prolonged follow-up is not a causal factor per se, its strong association with episode detection underscores the critical role of continuous rhythm monitoring in accurately identifying subclinical arrhythmias and characterizing their risk profile in this population.

Several working groups now recommend using a 90-day blanking period after device implantation to separate transient, inflammation-related episodes from true baseline rhythm. We therefore propose defining a new-onset AHRE as the first atrial episode ≥ 5-6 minutes at ≥ 175-190 bpm that occurs after day 90 post-implant, provided no AHRE ≥ 30 seconds is recorded during those initial three months. This 90-day threshold mirrors the lead-healing phase and the blanking period already endorsed in catheter-ablation consensus documents, where early arrhythmias are likewise attributed to procedural inflammation [1,44,45].

We recommend distinguishing early (90 days-12 months) from late (>12 months) AHRE onset and quantifying daily episode burden, as prognostic thresholds of six minutes, 5.5 hours, and 24 hours carry different clinical implications [46]. Explicit documentation of interrogation intervals, EGM-validation criteria, and any protocol deviations - such as a shorter blanking period with conduction-system pacing - will enhance cross-study comparability [47].

Electrocardiographic predictors

In our study, the electrocardiographic parameters collected were limited to QRS axis as an indirect indicator of lead position, and the presence or absence of left bundle branch block or IAB. No significant differences were observed between the study groups regarding these variables. However, multiple studies have emphasized the prognostic value of other ECG-derived predictors in detecting AHREs.

P-wave abnormalities on surface ECG and device-detected signals have been identified as significant predictors of AHREs. Reduced atrial sensing amplitude (<2.45 mV or ≤1.5 mV) is associated with an increased risk of both incident and progressive AHREs, including a linear decline in amplitude during the year preceding the first episode [10,31,48].

Prolonged PWD, particularly ≥160 ms in paced atrial activity or >100 ms on standard 12-lead ECG, has shown strong predictive value, especially in elderly patients [18,30,33]. Increased P-wave dispersion (PWDIS) and the presence of IAB (PWD >120 ms) have also been reported as independent predictors of AHREs [30,31]. Novel surface ECG markers, such as peak P-wave time in lead V1 and lead II, further enhance risk stratification [29,49].

Likewise, apical positioning of the right ventricular lead has been significantly associated with a higher incidence of AHREs [18]. Compared to left bundle branch area pacing (LBBAP) or septal right ventricular pacing (RVS), apical right ventricular pacing (RVA) is linked to longer paced QRS durations and higher AHRE incidence (36.4% vs. 12.5%) [18,50,51]. In contrast, LBBAP may reduce this risk by achieving a narrower QRS complex during implantation [52].

In sum, although our dataset revealed no inter-group differences in broad conduction indices (QRS axis, bundle-branch or IAB), the literature consistently identifies finer atrial-EGM markers - diminished sensing amplitude, prolonged or dispersed P-wave indices, refined peak-P timings - and ventricular lead geometry, particularly apical positioning with a widened paced QRS, as more robust electrocardiographic predictors of AHREs. Future investigations that integrate these high-resolution ECG parameters with precise lead-location data are warranted to enhance risk stratification and to inform pacing strategies aimed at mitigating device-detected atrial tachyarrhythmias.

Interpretation of the multivariable model

The loss of statistical significance observed for SSS in the multivariable analysis may be explained by its strong association with the AAI-DDD pacing mode, which is commonly used in this clinical context. AAI-DDD pacing is also closely linked to a lower ventricular pacing burden, a relationship that introduces potential confounding.

Although the VIF for low ventricular pacing (2.08) was just below our pre-specified exclusion threshold (VIF > 2.5), including this variable produced unstable coefficients and widened confidence intervals for AAI-DDD without improving model fit. Given its conceptual overlap with pacing mode and SSS, we excluded low ventricular pacing from the final model to preserve parsimony. All remaining predictors had VIF < 2, confirming acceptable collinearity.

Manufacturer was excluded from the multivariable model because several companies apply model-specific thresholds within the same brand; for example, Biotronik devices range from 180 to 220 bpm depending on the model, so “manufacturer” does not accurately capture the operative detection setting for each patient. This intra-brand variability, together with small counts in some brand-model subgroups, would introduce misclassification bias and unstable estimates; therefore, manufacturer was assessed only in univariate analysis and is now explicitly cited as a limitation, with a recommendation that future studies include larger, model-balanced samples or harmonized thresholds to examine manufacturer effects more robustly.

The multivariable logistic regression model demonstrated acceptable discriminative performance, with an area under the receiver operating characteristic curve (AUC) of 0.70, consistent with previous studies in this field. Although its predictive precision was acceptable, the model provided clinically relevant information for identifying patients at higher risk of AHREs and may support improved decision-making regarding individualized monitoring strategies and pacemaker programming optimization.

In device-based arrhythmia research, most published risk scores show comparable performance: for example, Chen et al. reported a model predicting de-novo AF in CIED carriers with an AUC of 0.806 [33], and the C2HEST (CAD or COPD (one point each), hypertension (one point), elderly (age ≥75 years, two points), systolic heart failure (two points), thyroid disease (one point)) score in predicting AHREs in patients with CIEDs without AF with an AUC of 0.73 [53]. Consequently, describing our value (AUC = 0.70) as acceptable places the model squarely within the range usually reported in the literature and avoids overstating its performance relative to comparable tools.

We acknowledge that the logistic regression model showed a modest discriminative performance (AUC = 0.70), which may reflect the inherent limitations of a retrospective design, the absence of detailed data on AHRE burden or duration, and the lack of structural atrial parameters such as atrial size, fibrosis markers, or advanced echocardiographic indices

Limitations

This study has several limitations, progressing from design to interpretation of results.

Retrospective, Observational, Single-Center Design

Because this is a retrospective observational study, all reported relationships are associative rather than causal, and prospective randomized evidence will be required to confirm any effect of pacing strategies on AHRE incidence. The study was conducted at a single specialized center in the Dominican Republic, which may limit the generalizability of the findings to other healthcare settings. Future research should incorporate prospective multicenter designs, standardized follow-up protocols, and comprehensive assessments of clinical and sociodemographic variables to validate and extend these results.

Sampling and Selection Bias

The consecutive inclusion of patients may have introduced sampling bias by preferentially capturing individuals with consistent follow-up and potentially under-representing those with lower adherence to clinical monitoring. Although only patients with complete datasets were included, this criterion may have introduced selection bias by excluding individuals with incomplete documentation or follow-up.

Incomplete Capture of Confounders

Important potential confounders, including treatment adherence and socioeconomic status, were not comprehensively assessed. Medication adherence and socioeconomic status were not systematically captured in our charts; nevertheless, future retrospective work could approximate these confounders through proxy measures such as insurance category (public vs. private), pharmacy-dispensing records or “proportion of days covered” for cardiovascular drugs, neighborhood deprivation indices derived from postal codes, and distance or travel time to the follow-up clinic. Incorporating these indirect indicators would allow sensitivity analyses for social- and adherence-related bias when direct metrics are absent.

Variable and Non-standardized Follow-Up

The retrospective nature of the study also precluded the prospective assessment of clinically relevant outcomes, such as stroke or systemic embolism associated with AHREs. Moreover, variability in follow-up duration introduced heterogeneity in episode detection, although this was partially addressed by stratifying patients into predefined temporal categories.

Episode Definition and Burden

Another key limitation is that all device-detected AHREs were included in the analysis regardless of episode duration, provided they were validated through expert EGM review. While this approach enhanced diagnostic specificity by excluding artefacts and non-arrhythmic signals, the absence of a minimum duration threshold may have led to an overestimation of AHRE prevalence. Short-lasting episodes, although morphologically consistent with atrial tachyarrhythmias, may have limited clinical relevance and uncertain prognostic value.

Episode duration and cumulative AHRE burden were not analyzed because device interrogations in this retrospective cohort occurred at non-standardized intervals, leaving episode-level metrics inconsistently documented and prone to measurement bias. We therefore treated AHREs dichotomously (present/absent) to preserve sample size and to meet our hypothesis-driven objective of identifying cross-sectional associations between clinical, electrocardiographic, and device-related variables and AHRE occurrence. While this approach maximizes analytical power, it restricts thromboembolic risk stratification and therapeutic inference, particularly regarding anticoagulation, because very brief, potentially benign episodes are evaluated alongside prolonged, clinically significant events. Future prospective studies employing continuous remote monitoring should capture duration and burden to validate these findings and refine risk-based management strategies.

Missing Anticoagulation Data

Because anticoagulation status (type, dose, adherence, and timing relative to AHRE detection) was not systematically recorded, we were unable to evaluate its potential effect modification. Future prospective registries should collect detailed anticoagulation data and stratify outcomes by both AHRE duration and CHA₂DS₂-VASc score, in line with recent NOAH-AFNET 6 and ARTESIA findings, to clarify therapeutic thresholds.

Device-Specific Technical Variability

Although significant differences in AHRE detection were observed across device manufacturers, this variable was excluded from the multivariable model, as it reflects inherent differences in diagnostic thresholds rather than true clinical or biological variation. As such, manufacturer-specific variability represents a technical limitation that affects diagnostic uniformity, rather than a factor causally related to AHRE development.

Statistical validation - Although internal robustness was checked with 1000-iteration bootstrapping, no k-fold cross-validation or external cohort testing was performed; future studies should apply k-fold cross-validation or out-of-sample bootstrapping and, where possible, replicate the model in multicentre datasets to estimate true generalisability.

Study strengths

This study offers several key strengths. It includes a clearly defined and adequately powered sample of 450 patients, reflecting real-world clinical practice in a low-resource setting. The objectives, inclusion criteria, and methodology were clearly stated and consistently applied. AHREs were validated by experienced electrophysiologists, ensuring diagnostic accuracy. Robust statistical analyses, including multivariable modeling with bootstrap validation, enhanced the reliability of findings. Finally, the study provides original data from a Latin American population, addressing a gap in the current literature on subclinical atrial arrhythmias.

Implications

Our results-41% AHRE prevalence and four easily obtainable predictors (prior AF/AT, OR 2.95; follow-up ≥ 90 days, OR 4.14; AAI-DDD mode, OR 1.92; female sex, OR 1.63)-have three practical implications: Patients carrying these factors should receive closer follow-up, including earlier remote monitoring, to detect subclinical arrhythmias; programming should be reviewed to avoid unnecessary atrial pacing and ensure an appropriate ventricular-pacing percentage, thereby mitigating an arrhythmogenic substrate; and the prediction model (AUC 0.70) can serve as an initial bedside tool to stratify thromboembolic risk and guide targeted AF screening and anticoagulation decisions.

Future research directions

Future investigations should adopt a comprehensive prospective framework. Continuous remote monitoring must record episode duration and cumulative AHRE burden, while linkage to stroke and mortality registries should provide hard end-points. Detailed documentation of anticoagulation status and adherence, augmented by proxy measures such as pharmacy-dispensing records and insurance category, will be essential to mitigate treatment and socioeconomic confounding. In parallel, the incorporation of inflammatory and profibrotic biomarkers (hs-CRP, IL-6, TNF-α, galectin-3, monocyte-to-HDL ratio) could illuminate causal pathways and refine risk prediction.

Building on the present findings, we propose a two-step research agenda. First, derive an AHRE-risk score that integrates the four independent predictors identified in this study (prior AF/AT, AAI-DDD mode, follow-up ≥90 days, female sex) with two established ECG variables (ventricular-pacing burden ≥50% and paced-QRS duration) and the arrhythmic metric itself-episode duration or total burden stratified into <6 minutes, six minutes-5.5 hours, 5.5 hours-24 hours, and >24 hours. This composite score would classify patients into low, intermediate, and high-risk categories.

Second, validate the score prospectively in a multicenter trial versus standard care, assessing incident and cumulative AHREs, thromboembolic events, anticoagulation utilization, and device longevity. Robust internal validation (k-fold cross-validation or bootstrapping) and external replication across diverse healthcare settings will be required to confirm generalizability.

This roadmap directly addresses current evidence gaps - episode burden, anticoagulation effect modification, external validation, and biomarker integration - and offers a structured pathway toward risk-stratified pacing strategies informed by both clinical and mechanistic data.

## Conclusions

In this cohort of patients with dual-chamber pacemakers, the prevalence of AHREs was 41.1%. AHREs occurred more frequently in female patients, in those with a history of AF or AT, in individuals with follow-up ≥ 90 days, and in devices programmed in AAI-DDD mode; a lower ventricular-pacing burden showed an additional association in bivariate analysis. Tailored pacing strategies were associated with a lower prevalence of AHREs in this cohort. Prospective randomized studies are needed to determine whether programming adjustments can actually reduce clinical arrhythmias or thromboembolic events. Our multivariable model showed moderate discrimination (AUC = 0.70) and good calibration, underscoring its potential as an initial risk-stratification tool.
